# Supplementary material for: Urinary Epidermal Growth Factor/Creatinine Ratio and Graft Failure in Renal Transplant Recipients: A Prospective Cohort Study
Source: J Clin Med. 2019 Oct 13;8(10):1673. doi: 10.3390/jcm8101673 (PMC6832301; doi:10.3390/jcm8101673)
Supplement: Supplementary file 1 [file jcm-08-01673-s001.zip › 23-07-STROBE EGF.docx]

STROBE Statement—Checklist of items that should be included in reports of ***cohort studies***

|  | Item No | Recommendation |
| --- | --- | --- |
| **Title and abstract** | 1 | (*a*) Indicate the study’s design with a commonly used term in the title or the abstract  Yes, study design is described in the tittle. |
|  |  | (*b*) Provide in the abstract an informative and balanced summary of what was done and what was found  Yes, in our abstract we included the most relevant information about background, study design, results and conclusions. |
| Introduction | | |
| Background/rationale | 2 | Explain the scientific background and rationale for the investigation being reported  Yes, we explained why graft failure is an important topic of study, presented the previous evidence discussing EGF and fibrosis in renal pathologies and addressed the gap of knowledge regarding post transplantation setting. |
| Objectives | 3 | State specific objectives, including any prespecified hypotheses  Yes, we described our main hypothesis that post-renal transplantation urinary EGF/creatinine ratio is associated with the risk of graft failure. Also, the secondary objectives of evaluation the predictive ability of this ratio |
| Methods | | |
| Study design | 4 | Present key elements of study design early in the paper  Yes, we extensively described the study design in the first subheading of the section “Materials and Methods”. |
| Setting | 5 | Describe the setting, locations, and relevant dates, including periods of recruitment, exposure, follow-up, and data collection  Yes, we described the setting and specified the dates and place of recruitment, the time of follow up and the date until data was collected and the mechanism used for collecting it. |
| Participants | 6 | (a) Give the eligibility criteria, and the sources and methods of selection of participants. Describe methods of follow-up  Yes, we described the inclusion criteria and how participants were selected and invited to participate, also the exclusion criteria. Likewise, we specified how the follow up was performed for all participants. |
|  |  | (*b*) For matched studies, give matching criteria and number of exposed and unexposed  Does not apply |
| Variables | 7 | Clearly define all outcomes, exposures, predictors, potential confounders, and effect modifiers. Give diagnostic criteria, if applicable  Yes, when necessary we gave clear definitions on which diagnosis criteria were used and how certain variables were calculated, the information can be found in the subheading ‘laboratory measurements and calculations’ |
| Data sources/ measurement | 8* | For each variable of interest, give sources of data and details of methods of assessment (measurement). Describe comparability of assessment methods if there is more than one group  Yes, we described the sources of data and the different measuring techniques used for obtaining it. The information can be found in the subheading ‘data collection’ and ‘laboratory measurements and calculations |
| Bias | 9 | Describe any efforts to address potential sources of bias  Yes, we described our strict research protocol, design to reduce bias. |
| Study size | 10 | Explain how the study size was arrived at  Yes, we explained hoy many patients were invited to participate and what exclusions were made until the final study size was reached |
| Quantitative variables | 11 | Explain how quantitative variables were handled in the analyses. If applicable, describe which groupings were chosen and why  Yes, we described when a continuous variable was transformed into a categorical variable (*e.g* tertiles). Explanations were given for the transformation and the cut-off values used were reported. |
| Statistical methods | 12 | (*a*) Describe all statistical methods, including those used to control for confounding  Yes, we described the method we use to compare subgroups in the baseline characteristics table, to perform the prospective analyses and to correct for potential confounders. |
|  |  | (*b*) Describe any methods used to examine subgroups and interactions  Yes, we described how we performed the interaction analyses and results are enclosed in the supplemental material. |
|  |  | (*c*) Explain how missing data were addressed  Yes, we explained which patients were excluded because of missing data |
|  |  | (*d*) If applicable, explain how loss to follow-up was addressed  Does not apply |
|  |  | (*e*) Describe any sensitivity analyses  Yes, we described the secondary analyses performed to test the strength of the association for our main outcome, and results are found in the supplemental material. |
| Results | | |
| Participants | 13* | (a) Report numbers of individuals at each stage of study—eg numbers potentially eligible, examined for eligibility, confirmed eligible, included in the study, completing follow-up, and analysed  Yes, we described the number of individuals at every stage and the number finally used for analyses. |
|  |  | (b) Give reasons for non-participation at each stage  Yes, we described the reasons for exclusion of participants. |
|  |  | (c) Consider use of a flow diagram  Does not apply |
| Descriptive data | 14* | (a) Give characteristics of study participants (eg demographic, clinical, social) and information on exposures and potential confounders  Yes, information available in the baseline characteristics table (table 1) |
|  |  | (b) Indicate number of participants with missing data for each variable of interest  Yes, information available for every variable in the baseline characteristic table (table 1) |
|  |  | (c) Summarise follow-up time (eg, average and total amount)  We had a settled follow-up time of 3 years. |
| Outcome data | 15* | Report numbers of outcome events or summary measures over time  Yes, we reported the number of events for every outcome in the follow-up. When subgroups analyses were performed, we also clarified the number of outcomes in each subgroup. |
| Main results | 16 | (*a*) Give unadjusted estimates and, if applicable, confounder-adjusted estimates and their precision (eg, 95% confidence interval). Make clear which confounders were adjusted for and why they were included  Yes, we presented a crude analysis first and then adjusted for potential confounders, the potential confounders used in the models of adjustment were specified and we clarify the criteria used to choose them. |
|  |  | (*b*) Report category boundaries when continuous variables were categorized  Yes, we described the boundaries of uEGF/cr ratio when we categorized to tertiles. |
|  |  | (*c*) If relevant, consider translating estimates of relative risk into absolute risk for a meaningful time period  Does not apply. |
| Other analyses | 17 | Report other analyses done—eg analyses of subgroups and interactions, and sensitivity analyses  Yes, the results of our interaction and sensitivity analyses are reported in the supplemental material. |
| Discussion | | |
| Key results | 18 | Summarise key results with reference to study objectives  Yes, we started the discussion by summarizing the main finding that answers our hypothesis. |
| Limitations | 19 | Discuss limitations of the study, considering sources of potential bias or imprecision. Discuss both direction and magnitude of any potential bias  Yes, we described the limitations of our study in the final part of the discussion. |
| Interpretation | 20 | Give a cautious overall interpretation of results considering objectives, limitations, multiplicity of analyses, results from similar studies, and other relevant evidence  Yes, we discussed our results taking into account the previous evidence and how our findings relate to it. |
| Generalisability | 21 | Discuss the generalisability (external validity) of the study results  Yes, in the discussion we addressed how the characteristics of our sample differed from the general population and how this affected generalization. |
| Other information | | |
| Funding | 22 | Give the source of funding and the role of the funders for the present study and, if applicable, for the original study on which the present article is based  Yes, we described the sources of funding for the cohort study in which this paper is based. |

*Give information separately for exposed and unexposed groups.

**Note:** An Explanation and Elaboration article discusses each checklist item and gives methodological background and published examples of transparent reporting. The STROBE checklist is best used in conjunction with this article (freely available on the Web sites of PLoS Medicine at http://www.plosmedicine.org/, Annals of Internal Medicine at http://www.annals.org/, and Epidemiology at http://www.epidem.com/). Information on the STROBE Initiative is available at http://www.strobe-statement.org.
