# Supplementary material for: Urinary Epidermal Growth Factor/Creatinine Ratio and Graft Failure in Renal Transplant Recipients: A Prospective Cohort Study
Source: J Clin Med. 2019 Oct 13;8(10):1673. doi: 10.3390/jcm8101673 (PMC6832301; doi:10.3390/jcm8101673)
Supplement: Supplementary file 1 [file jcm-08-01673-s001.zip › 23-07 EGF and GF-Supplemental material-JCM.docx]

*Supplemental material*

Urinary epidermal growth factor/creatinine ratio and graft failure in renal transplant recipients: a prospective cohort study.

Manuela Yepes-Calderón ^1^, Camilo G. Sotomayor ^1,^*, Matthias Kretzler^2^, Rijk O.B. Gans ^3^, Stefan P. Berger^1^, Gerjan J. Navis^1^, Wenjun Ju^2^ and Stephan J.L. Bakker^1^.

^1^ Division of Nephrology, Department of Internal Medicine, University Medical Center Groningen, University of Groningen, Groningen, The Netherlands.

^2^ Department of Internal Medicine, Department of Computational Medicine and Bioinformatics, University of Michigan, Michigan, Unite States of America.

^3^ Department of Internal Medicine, University Medical Center Groningen, University of Groningen, Groningen, The Netherlands.

***** Correspondence: c.g.sotomayor.campos@umcg.nl; Tel.: 0031 061 925 27 12

Received: date; Accepted: date; Published: date

1. Table S1

**Table S1.** Comparison of baseline characteristics between renal transplant recipients with and without data of urinary EGF concentration.

| Baseline characteristics | | |  | RTR included in the final analyses |  | RTR with missing uEGF values |  | | *P* value |
| --- | --- | --- | --- | --- | --- | --- | --- | --- | --- |
|  |  | |  | *n*=649 |  | *n*=57 |  | |  |
|  | Age, years | |  | 53 ± 13^†^ |  | 53 ± 13 |  | | 0.96 |
|  | Sex, male | |  | 373 (58) ^§^ |  | 28 (49) |  | | 0.22 |
|  | eGFR, mL/min/1.73 m^2^ | |  | 52 ± 20 |  | 50 ± 21 |  | | 0.40 |
|  | Urinary protein excretion, g/24 h | |  | 0.20 [0.02−0.34] ^‡^ |  | 0.20 [0.02−0.55] |  | | 0.27 |
|  | Pre−emptive transplant, *n* (%) | |  | 105 (16) |  | 8 (14) |  | | 0.67 |
|  | Age of donor, years | |  | 43 ± 15 |  | 41 ± 17 |  | | 0.27 |
|  | Sex of donor (male) , *n* (%) | |  | 331 (51) |  | 26 (46) |  | | 0.50 |
|  | Time since transplantation, years | |  | 5.28 [1.74−12.00] |  | 7.00 [3.14−12.15] |  | | 0.20 |
|  | Cumulative prednisolone dose, g | |  | 17.4 [5.2−36.2] |  | 19.3 [7.7−35.1] |  | | 0.22 |
|  | Sirolimus or rapamune use, *n* (%) | |  | 13 (2) |  | 1 (2) |  | | 0.27 |
|  | Type of calcineurin inhibitor | | | | | | |  |  |
|  |  | Cyclosporine, *n* (%) |  | 258 (40) |  | 19 (33) |  | | 0.34 |
|  |  | Tacrolimus, *n* (%) |  | 120 (19) |  | 10 (18) |  | | 0.86 |
|  | Type of proliferation inhibitor | | | | | | |  |  |
|  |  | Mycophenolic acid, *n* (%) |  | 424 (65) |  | 39 (68) |  | | 0.64 |
|  |  | Azathioprine, *n* (%) |  | 112 (17) |  | 11 (19) |  | | 0.70 |
|  | Acute rejection treatment, *n* (%) | |  | 172 (27) |  | 17 (30) |  | | 0.59 |

Abbreviations: eGFR, estimated glomerular filtration rate. Differences were tested by ANOVA or Kruskal-Wallis test for continuous variables and by χ2 test for categorical variables. †Mean ± (standard deviation); §n (percentage); ‡Median [interquartile range], all such values.

2. Table S2

**Table S2.** Effect-modification of pre-specified baseline characteristics on the associations of uEGF/Cr with graft failure.

|  |  | Graft failure | |
| --- | --- | --- | --- |
|  |  | B | *P* |
| Age, years |  | 0.004 | 0.53 |
| Sex |  | -0.09 | 0.50 |
| eGFR, ml/min/m^2^ |  | 0.003 | 0.16 |
| Creatinine |  | 0.00 | 0.87 |
| Urinary protein excretion, g/24h |  | -0.06 | 0.58 |
| hsCRP, mg/L |  | 0.00 | 0.96 |
| Acute rejection |  | -0.23 | 0.19 |
| Pre-emptive transplantation |  | -0.30 | 0.51 |

Cox-proportional hazards regression analysis was performed to assess multiplicative interaction terms. B, standardized beta coefficient.

3. Table S3

**Table S3.** Multivariable-adjusted associations between uEGF/Cr ratio and graft failure in RTR among the -2 and +2 standard deviations of uEGF/Cr.

|  |  | HR per ng/mg | 95% CI |  | *P* value |
| --- | --- | --- | --- | --- | --- |
| Model 1 |  | 0.68 | 0.59-0.78 |  | <0.001 |
| Model 2 |  | 0.67 | 0.57-0.77 |  | <0.001 |
| Model 3 |  | 0.67 | 0.57-0.78 |  | <0.001 |
| Model 4 |  | 0.78 | 0.66-0.93 |  | 0.005 |

In total 630 patients had uEGF/Cr between -2 and +2 standard deviations. 40 (6%) patients developed graft failure. Model 1: crude. Model 2: model 1 + age and sex, and transplant related data. Model 3: model 1 +age and sex, and immunosuppressive therapy. Model 4: model 1 +age and sex, and graft function

| 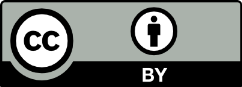 | © 2019 by the authors. Submitted for possible open access publication under the terms and conditions of the Creative Commons Attribution (CC BY) license (http://creativecommons.org/licenses/by/4.0/). |
| --- | --- |
